# *Akkermansia muciniphila* NND9 Mitigates Ulcerative Colitis by Ameliorating the Gut Barrier *via* Suppressing DR5 Expression in a Mouse Model

**DOI:** 10.3390/microorganisms14051002

**Published:** 2026-04-29

**Authors:** Xin-Yu Gao, Yan Wang, Yu-Hui Wang, Hao Yu, Liang Liu, Xing-Hua Zhang, Hong-Tao Xu, Yao Meng, Randal N. Johnston, Gui-Rong Liu, Shu-Lin Liu

**Affiliations:** 1 Genomics Research Center (Key Laboratory of Gut Microbiota and Pharmacogenomics of Heilongjiang Province, and State-Province Key Laboratory of Biomedicine-Pharmaceutics of China), College of Pharmacy, Harbin Medical University, Harbin 150081, China; gxy18943331136@163.com (X.-Y.G.);; 2 National Key Laboratory of Frigid Zone Cardiovascular Diseases, Harbin Medical University, Harbin 150081, China; 3 Department of Biochemistry and Molecular Biology, University of Calgary, Calgary, AB T2N 4N1, Canada; 4 Department of Microbiology, Immunology and Infectious Diseases, University of Calgary, Calgary, AB T2N 4N1, Canada

**Keywords:** ulcerative colitis, *Akkermansia muciniphila*, gut barrier, the gut microbiome, DR5

## Abstract

Ulcerative colitis (UC) is a type of inflammatory bowel disease without curative therapeutics. Recent studies demonstrate that *Akkermansia muciniphila* exerts mitigating effects on UC, but the underlying mechanisms remain unclear. In this study, we isolated a strain of *A. muciniphila*, designated NND9, from the feces of DSS-induced ulcerative colitis model mice and investigated its effects on UC of the mouse model. NND9 significantly alleviated UC severity in the mice by restoring gut barrier integrity through improving colonic mucus layer thickness, mitigating goblet cell depletion, and halting epithelial cell death. Mechanistically, NND9 suppressed the expression of the *Tnfrsf10b* gene encoding death receptor 5 (DR5) on the surface of colonic epithelial cells. Additionally, NND9 inhibited the phosphorylation of kinase 3 (RIPK3) and the pseudokinase mixed-lineage kinase domain-like protein (MLKL) associated with the necrotic apoptosis pathway, thereby reducing gut epithelial cell death. NND9 also markedly ameliorated the gut microbiome of the colitis mice. Untargeted metabolomics analysis demonstrated that NND9 modulated both tryptophan and bile acid metabolism. In conclusion, NND9 exhibits curative effects on UC by resolving inflammatory reactions of the gut mucosa through the DR5-RIPK3/p-RIPK3-MLKL/p-MLKL pathway and redressing gut dysbiosis. This study provides valuable information for the development of innovative therapeutic strategies for the treatment of UC.

## 1. Introduction

Ulcerative colitis (UC) is a type of inflammatory bowel disease (IBD) affecting the colon. The characteristic manifestations include diarrhea, bloody stools, and weight loss, with episodes alternating between flare-ups and remissions [1,2,3,4,5]. The etiology remains unclear, so clinical treatments primarily consist of symptomatic therapies [6], depending on the severity of the disease and the extent of colon involvement. Novel treatment approaches are emerging, such as those modulating the gut immune system, accelerating repair of the gut epithelial barrier, and using probiotic formulations to restore the disrupted gut ecosystem [7]. However, curative therapies that aim at resolving the inflammation rather than merely mitigating the symptoms are still a notional goal to be achieved.

Extensive research indicates that gut dysbiosis is closely associated with IBD, which has been repeatedly confirmed in mouse models, defined as a marked reduction in gut microbial diversity and massively altered balance among microbes of different phylogenetic groupings [8]. Studies have further revealed that certain gut microbes and their metabolites play crucial roles in IBD pathogenesis or disease remission by modulating immune responses, damaging or enhancing the gut epithelial barrier, and eliciting or resolving inflammation [9,10]. *Akkermansia muciniphila* is a representative bacterial species of the phylum Verrucomicrobiota present in the normal human gut microbiota as resident bacteria [11]. Numerous reports have demonstrated that *A. muciniphila* can alleviate colitis. For instance, *A. muciniphila* type strain ATCC BAA-835 exerts alleviative effects in colitis model mice by modulating gut microbiota composition, restoring the gut barrier, and affecting the production of host metabolites; additionally, *A. muciniphila* can stimulate immune cells and coordinate anti-inflammatory responses [12,13,14,15].

The gut barrier comprises an external mucus layer and the epithelial layer, serving as the first line of defense for the mucosal immune system against diseases such as UC [16]. *A. muciniphila* can restore mucus layer thickness by increasing mucin production and may participate in mucin subtype modification [17]. Additionally, *A. muciniphila* promotes gut homeostasis by enhancing epithelial cell development through stem cell proliferation [18]. However, the roles of *A. muciniphila* in repairing the gut epithelial layer and the underlying mechanisms during chronic UC remain largely unclear.

Death receptor 5 (DR5), also known as TNF-related apoptosis-inducing ligand receptor 2 (TRAIL-R2), is a member of the tumor necrosis factor receptor superfamily (TNFRSF) and is widely expressed at low levels on the cell membrane as a transmembrane receptor [19,20]. Activation of DR5 induces cell death by either apoptosis or necrosis, or combined programmed cell death processes [21,22]. In normal cells, overexpression of the DR5 protein could disrupt the balance between cell survival and death, leading to excessive cell death. Necrotic apoptosis, a special form of cell death [23,24], depends on the activation of receptors within the TNFRSF subfamily, including TNFR1, Fas, and DR5 [25].

In this study, we characterized a fresh isolate of *A. muciniphila* from mouse feces, designated as strain NND9, which showed a protective effect on the gut epithelial barrier to mitigate colitis in DSS-induced UC mice. We found that *A. muciniphila* NND9 significantly suppressed the expression of DR5 in the colon tissues of the experimental UC mice, thereby reducing gut epithelial cell death, restoring gut barrier function, and regaining gut homeostasis. Our findings suggest that DR5 could be a therapeutic target for chronic colitis, providing a new way to treat the disease.

## 2. Materials and Methods

### 2.1. Isolation and Identification of A. muciniphila

Fresh feces were collected from SPF-grade male BALB/c mice with DSS-induced UC. The fecal samples were placed in tubes containing 50% glycerol, mixed thoroughly, and stored at −80 °C prior to use. The fecal suspension was serially diluted with sterile water to a final dilution of 10^−3^ concentration. The diluted fecal suspension was inoculated onto 0.4% porcine type II mucin (Sigma, M2378, St. Louis, MO, USA) agar plates without carbon sources. The plates were incubated at 37 °C in an anaerobic chamber (10% H_2_, 10% CO_2_, 80% N_2_) for 48–72 h until distinct colonies formed. Milky-white single colonies were picked up and streaked on mucin agar plates for purification. The isolated strain’s gene sequence was then amplified by PCR using specific primers: AM1 (5′-CAGCACGTGAAGGTGGGGAC-3′) and AM2 (5′-CCTTGCGGTTGGCTTCAGAT-3′) [26]. PCR products were analyzed by electrophoresis on a 1% agarose gel, and positive strains were selected based on band position (~327 bp). Five positive strains were selected for PCR amplification using universal 16S primers 27F (5′-AGAGTTTGATCCTGGCCTCA-3′) and 1492R (5′-GGTTACCTTGTTACGACTT-3′). Positive PCR products underwent 16S rDNA sequencing (Genesoul Technology, Harbin, China). Sequence alignment of the five positive strains in the NCBI database identified them as *Akkermansia* strains. After 48 h of cultivation in BHI liquid medium containing 0.4% mucin, the bacterial culture was collected and mixed with 50% autoclaved glycerol (1:1, *v*/*v*). The mixture was stored overnight at −20 °C before transfer to −80 °C for long-term preservation.

### 2.2. Whole-Genome Sequencing of A. muciniphila Strain NND9

The strain NND9, which was preliminarily identified as *A. muciniphila*, was cultured in BHI liquid medium containing mucin and cysteine. The bacterial cells in the culture were collected by centrifuging at 4 °C, 10,000 rpm for 5 min. The genomic DNA of strain NND9 was initially extracted using the Invitrogen PureLink^®^ Genomic DNA kit (Omega Bio-Tek, Norcross, GA, USA). The DNA quantity and quality were tested by the NanoDrop ND-1000 Spectrophotometer (Thermo Fisher Scientific, Waltham, MA, USA). The DNA was purified further using the Quick-DNA Miniprep Plus kit. Whole genome sequencing was performed by Pacific Biosciences Sequel II technology (PacBio, Shanghai, China).

### 2.3. Culture and Preparation of A. muciniphila NND9

*A. muciniphila* NND9 was cultured on Brain Heart Infusion (BHI) plates (LAND BRIDGE, Beijing, China) containing 0.4% porcine type II mucin (Sigma) and 0.05% L-cysteine (Macklin, Shanghai, China) and incubated in a 37 °C anaerobic chamber (10% H_2_, 10% CO_2_, and 80% N_2_) for 48 h. The relationship between bacterial density and OD value was determined as OD_600_ = 1.5 was equivalent to 1 × 10^9^ CFU/mL bacteria. NND9 was cultured on agar plates in anaerobic incubators for 48–72 h. Prior to each gavage, bacterial suspensions were prepared using autoclaved PBS to achieve an OD_600_ = 1.5, and 300 μL of bacterial suspension was administered to each mouse.

### 2.4. Scanning Electron Microscopy (SEM)

The bacterial density was adjusted using autoclaved PBS buffer to an OD_600_ of 1.5. One milliliter of the bacterial suspension was transferred to a 1.5 mL centrifuge tube and centrifuged to collect the bacterial pellet (12,000× *g*, RT). The supernatant was discarded, and 2.5% glutaraldehyde fixative was added slowly. The sample was fixed at RT for 1–2 h and then at 4 °C overnight. After three 5 min PBS washes, they were fixed with 1% osmium tetroxide for 1 h and washed thrice more. The sample was dehydrated using graded ethanol (30%, 50%, 70%, 90%; 15 min each), and then treated with 100% ethanol for 20 min and transferred to fresh 100% ethanol. After critical point drying, the samples were mounted on the stage with conductive carbon glue, sputter-coated with Pt for 70 s, and then imaged by SEM.

### 2.5. Animal Experiments

Six seven-week-old SPF-grade male BALB/c mice (20–22 g) were purchased from Beijing Vital River Laboratory Animal Technology Co., Ltd. (Beijing, China). The mice were housed in a specific pathogen-free animal facility maintained at controlled temperature and humidity conditions under 12 h light-dark cycles, with free access to water and food. The mice were acclimated to the housing environment for 7 days prior to formal experimentation. After the adaptation phase, the mice were randomly assigned to three groups (6 mice/group): Control, DSS-induced UC model, and NND9 administration. Mice of the control group had free access to sterile water. Mice in the other two groups had free access to 2.2% sodium dextran sulfate (DSS, 36–50 kD, *w*/*v*) (MP Biomedicals, # 0216011080, Burlington, ON, Canada) prepared in sterile water for 6 days, followed by 10 days of free access to sterile water, constituting one cycle. Three cycles were performed to induce chronic ulcerative colitis in the mice. Starting on day 6, the mice in the NND9 group received 300 μL of bacterial suspension prepared in PBS via gavage every day. Concurrently, mice in the CON and DSS groups received 300 μL of PBS via gavage every day until the end of the experiment. The mice were anesthetized with tribromoethanol, blood was collected, and the mice were euthanized. All animal procedures were approved by the Animal Ethics Committee of Harbin Medical University (Ethics License No. HMUIRB2026001).

### 2.6. Animal Sample Collection

The mice were euthanized and then dissected to collect tissues. Briefly, the colon was removed for length measurement. A portion of the colonic tissue was fixed in 4% paraformaldehyde for histological staining, and the remainder was stored at −80 °C. The spleen was harvested and weighed to assess splenomegaly. The liver was also removed and weighed. A portion of the liver tissue was fixed for H&E staining for histopathological examination, while the rest was stored at −80 °C.

### 2.7. Histological Staining

Fresh colon tissue was thoroughly rinsed in saline on ice. A 4–5 cm segment of distal colon tissue was obtained, fixed in 4% paraformaldehyde tissue fixative, embedded in paraffin, and sectioned into 3 μm thick slices. The same treatment was applied to the liver tissue. The sections underwent H&E staining, AB-PAS staining, and HID-AB staining. In brief, for H&E staining, paraffin sections of colon and liver tissues were stained using the Hematoxylin and Eosin (H&E) Pan-stable Staining Kit (Rena-Panaso, HJ-H0750-4, Zhuhai, China). Paraffin sections of colon tissue were, respectively, stained using the AB-PAS staining kit (Solarbio, Cat# G1285, Beijing, China) and the HID-AB staining kit (Solarbio, Cat# G2070) for AB-PAS staining and HID-AB staining. Positive cells within crypts were counted across randomly selected fields of view, and their numbers were compared among different tissue samples.

### 2.8. Assessment of Histological Score

The histopathological scoring was performed according to Table 1.

Different sections of the colon tissue were selected, and each parameter was calculated and summed for mice in each group to obtain a total score.

### 2.9. Immunohistochemical Staining

The paraffin sections were treated with the following primary antibodies, respectively, for immunohistochemistry: anti-MUC2 (1:500, HUABIO, ET1704-06, Hangzhou, China), anti-KI67 (1:500, HUABIO, HA721115) at 4 °C overnight. The slices were incubated with the HRP-conjugated secondary antibody (Dako, # K5007) for 50 min at RT the next day. Then, the slides were exposed to the diaminobenzidine solution (DAB, Dako, # K5007) to detect target signals and finally examined under a microscope for image capture. For MUC2 staining, quantification of the mucosal thickness was performed using Image J 1.53a. For KI67 staining, positive cells within crypts were counted across randomly selected fields of view, and their numbers were compared among different tissue samples.

### 2.10. Immunofluorescence Staining

For immunofluorescence, the sections were treated at 4 °C overnight with the primary antibody: anti-ZO-1 (1:100, Proteintech, Cat# 21773-1-AP, Wuhan, China) and anti-Occludin (1:100, HUABIO, Cat# ET1701-76), followed by incubation with CY3-labeled secondary antibody (1:100, Dako, Cat# K5007) at room temperature for 50 min. After PBS washing, the slices were mounted using 4′,6- diamidino-2-phenylindole (DAPI, Beyotime, C1002, Shanghai, China). A fluorescent microscope was used to examine and take pictures. The fluorescence intensity of positive cells and DAPI in different fields of view of colon tissue was separately quantified using ImageJ2 Fiji software. The ratio of fluorescence intensity (positive cells/DAPI) was calculated, and the relative fluorescence intensity among different tissue samples was compared.

### 2.11. MPO Assay

The collected mouse whole blood was allowed to stand at room temperature for 1 h and then centrifuged (4 °C, 3000 rpm, 10 min). The serum was aspirated, divided into portions, and stored at −80 °C. Colon tissue was weighed, placed in pre-chilled PBS (1:9, *m*/*v*), and homogenized in a tissue grinder at 4 °C. After thorough homogenization, the homogenate was centrifuged (4 °C, 3000 rpm, 20 min), and the supernatant was stored at −80 °C. MPO levels in serum and colon tissue supernatant were measured using an ELISA kit (MEIMIAN, Dalian, China, Cat# MM-0338M1) according to the manufacturer’s instructions.

### 2.12. RNA Extraction and Quantitative Real-Time Polymerase Chain Reaction (qRT-PCR)

Total RNA was extracted from the colon tissue and cells using FreeZol Reagent (Vazyme, Shanghai, China, Cat# R711-01) according to the manufacturer’s instructions. Messenger RNA was separated from total RNA using the Poly (A) enrichment method. The RNA concentration was measured, and cDNA was synthesized via reverse transcription using the Evo M-MLV RT Mix Kit with cDNA Clean for qPCR (Accurate Biology, Dalian, China, No. AG11728) following the protocol. Subsequently, specific primers were designed (Table 2), and polymerase chain reaction was performed using the SYBR Green Premix Pro Taq HS qPCR Kit (Accurate Biology, China, Cat# AG11701). ACTB was used as the internal reference gene, and the relative expression levels of mRNA in each sample were analyzed using the 2^−ΔΔCT^ method.

### 2.13. Western Blotting

Colon tissue was weighed, placed in tissue lysis buffer (1:10) containing protease inhibitor and phosphatase inhibitor (RIPA: protease inhibitor: phosphatase inhibitor = 100:1:1, *v*/*v*/*v*), homogenized in a tissue grinder at 4 °C, allowed to stand for 30 min, and then centrifuged, and the protein supernatant was collected after centrifugation (12,000 rpm, 10 min). The protein concentration was determined using the BCA method. The protein samples were separated by electrophoresis on a 10% SDS polyacrylamide gel and transferred to polyvinylidene fluoride (PVDF) membranes. Unoccupied sites on the PVDF membranes were blocked using Rapid Blocking Solution (Servicebio, Wuhan, China, Cat# G2052-500ML). Primary antibodies against DR5 (Cat#A96648), Ripk3 (Cat#68786-2-Ig), Phospho-Ripk3 (Cat#bsm-62802R), MLKL (Cat#RC-7050), or Phospho-MLKL (Cat#bsm-54104R) were added. Protein samples and primary antibodies were incubated overnight at 4 °C. After washing the PVDF membranes, HRP-conjugated secondary antibodies were added, and the membranes were incubated at room temperature for 1–2 h. The protein bands were visualized using an ultra-sensitive ECL chemiluminescent substrate (Meilun, Dalian, China, MA0186-2). β-actin served as the internal control protein and the relative protein expression levels were quantified using ImageJ.

### 2.14. RNA Sequencing and Analysis

Total RNA was extracted from mouse colon tissue using TRIzol^®^ reagent (Majorbio, Shanghai, China) according to the manufacturer’s instructions. Messenger RNA was isolated according to the polyA selection method by oligo (dT) beads. RNA purification, reverse transcription, library construction, and sequencing were performed at Shanghai Majorbio Bio-pharm Biotechnology Co., Ltd. (Shanghai, China) according to the manufacturer’s instructions (Illumina, San Diego, CA, USA). Using the NovaSeq Xplus platform (Illumina, San Diego, CA, USA), all mRNAs transcribed from the mice colon tissue were sequenced. Data analysis was performed using the Majorbio Cloud (www.majorbio.com, accessed on 1 August 2025). Differential expression analysis was performed using DESeq2 or DEGseq. Genes with |log_2_ FC| ≥ 1 and FDR ≤ 0.05 (DESeq2) or FDR ≤ 0.001 (DEGseq) were deemed significantly differentially expressed genes (DEGs). Consequently, volcano plot, gene expression heat map, and KEGG pathway analysis of these DEGs were performed.

### 2.15. Cell Culture and Treatment

The human colon mucosal epithelial cell line NCM460 was cultured in RPMI1640 (Gibco, CA, USA, C11875500BT) supplemented with 10% Premium Grade Fetal Bovine Serum (FBS, ENTROL, Cat#ESFBS50, China). All cells were grown at 5% CO_2_ and 37 °C. The DR5 overexpression plasmid (GenePharma, Shanghai, China) was constructed, and the NCM460 cells were transfected with the GP-transfect-Mate transfection reagent (GenePharma, G04008) according to the manufacturer’s instructions. After transfection for 24 h, the cells were subsequently treated for 24 h with the empty vector (Negative Control, NC), the DR5 overexpression plasmid (DR5^+^), or DR5 together with NND9 (DR5^+^ + NND9). The ratio of cells to the bacterial strain NND9 was 1:10.

### 2.16. Cell Viability Test

NCM460 cells were seeded into a 96-well plate at a density of 7500 cells per well and divided into four groups: the NC group, the DR5^+^ group, the DR5^+^ + NND9 group, and the NC + NND9 group. Transfection of NCM460 cells was performed according to the aforementioned cell treatment method. After transfection for 24 h, NND9 was added at a ratio of 10:1 (bacteria:cell). The cells were cultured for another 24 h. After that, the old medium was removed, and a mixture of CCK8 solution and RPMI1640 medium at a ratio of 1:9 was added to the cells, with 100 μL of the culture medium added to each well. The plates were incubated in a 37 °C cell incubator under dark conditions for 2 h. The optical density (OD) was measured at 450 nm using a microplate reader. The experiment was repeated three times.

### 2.17. Annexin V-FITC/Propidium Iodide (PI) Assay

NCM460 cells subjected to different treatments were fluorescently stained using the Annexin V-FITC/PI Apoptosis Detection Kit (MeilunBio, Dalian, China, Cat# MA0220) according to the manufacturer’s protocol. In brief, early apoptotic cells were identified as Annexin V-positive (Annexin V+/PI−), while late apoptotic and necrotic cells were double-stained with both FITC and PI (Annexin V+/PI+).

### 2.18. Fecal 16S rDNA Microbial Community Analysis

Fresh feces from mice in different groups were collected, immediately placed in sterile tubes, and stored at −80 °C. Fecal samples from each group underwent total genomic DNA extraction for microbial communities using the FastPure Stool DNA Isolation Kit (MJYH, Shanghai, China) according to the manufacturer’s instructions. The extracted DNA served as a template for PCR amplification of the 16S rDNA gene V3-V4 variable region using barcoded primers 338F (5′-ACTCCTACGGGAGGCAGCAG-3′) and 806R (5′-GGACTACHVGGGTWTCTAAT-3′). PCR products were recovered from 2% agarose gels, purified, and quantified using the Synergy HTX (Biotek, Vinuschi, VT, USA). The purified PCR products were library-prepared using the NEXTFLEX Rapid DNA-Seq Kit (Bioo Scientific, Austin, TX, USA). Sequencing was performed on the Illumina NextSeq 2000 PE300 platform (Majorbio, Shanghai, China). Bioinformatic analysis of the gut microbiota was carried out using the Majorbio Cloud platform (https://cloud.majorbio.com, accessed on 1 August 2025).

The alpha diversity indices, including observed OTUs, ACE index, Chao richness, and Shannon index, were calculated using Mothur v1.30.2. The similarity of microbial communities in different samples was measured by PCoA based on Bray–Curtis dissimilarity using the Vegan v2.4.3 package. The LEfSe was performed to identify the significantly abundant taxa (phyla to genera) of bacteria among the different groups (LDA score > 2, *p* < 0.05).

### 2.19. LC-MS Untargeted Metabolomics Analysis of Mouse Feces

Fresh feces from mice of different groups were collected, immediately placed in sterile tubes, and stored at −80 °C. For fecal metabolite composition analysis, 50 mg solid sample was extracted with 400 μL of extraction solvent (methanol: water = 4:1 (*v*/*v*)) containing 0.02 mg/mL internal standard (L-2-chlorophenylalanine) to obtain metabolites. The sample suspension was ground for 6 min in a cryogenic tissue grinder (−10 °C, 50 Hz), followed by low-temperature ultrasonic extraction for 30 min (5 °C, 40 kHz). The sample was allowed to settle at −20 °C for 30 min, then centrifuged for 15 min (4 °C, 13,000× *g*). The supernatant was transferred to an injection vial with an insert tube for instrument analysis. Mass spectrometry data were matched against public metabolite databases, including HMDB, Metlin, and the Majorbio database. Metabolite information was obtained by identifying metabolites through database searches. The data were analyzed through the free online platform of the Majorbio cloud platform (cloud.majorbio.com, accessed on 1 August 2025). The metabolites with VIP > 1, *p* < 0.05 were determined as significantly different metabolites based on the Variable importance in the projection (VIP) obtained by the OPLS-DA model and the *p*-value generated by Student’s *t* test. These metabolites were annotated for metabolic pathways using the KEGG database. The Python package “scipy. stats” (https://docs.scipy.org/doc/scipy/, accessed on 9 February 2025) was used to perform enrichment analysis.

### 2.20. Statistical Analysis

The statistical analysis software GraphPad Prism 5.0 was used for data analysis and visualization. A one-way ANOVA was employed to assess the statistical significance of differences among groups. The data are presented as mean ± standard deviation (SD). A *p*-value < 0.05 indicates statistical significance (* *p* < 0.05, ** *p* < 0.01, *** *p* < 0.001, **** *p* ≤ 0.0001), and ns denotes no significant difference.

## 3. Results

### 3.1. Morphological and Genomic Characteristics of A. muciniphila NND9

**Figure 1:**
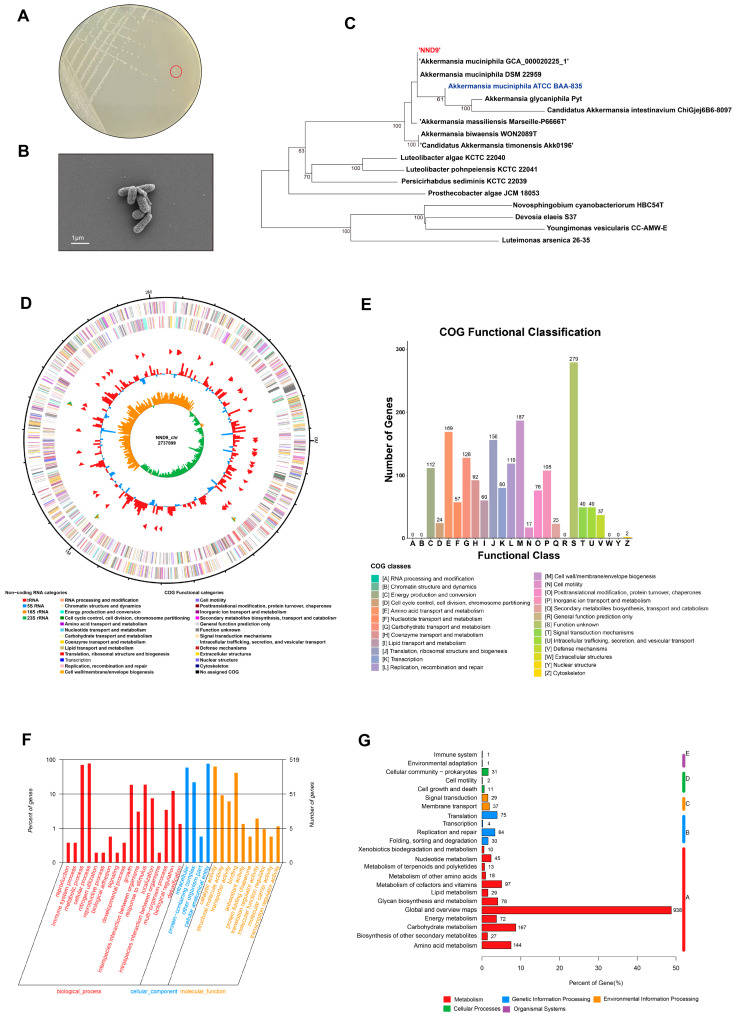
Morphological characteristics and whole-genome sequence analysis of NND9. (**A**) Colony morphology of strain NND9 on BHI agar plates containing 0.4% mucin. (**B**) Scanning electron micrograph (SEM) of NND9. Bar, 1 mm. (**C**) Phylogenetic relationships of NND9 with closely related bacteria based on 16S rDNA gene sequences. (**D**) Genome map of strain NND9. From outer to inner: (1) Genome size labeling; (2) CDS on the positive strand; (3) CDS on the negative strand, with different colors representing the COG functional classification of the CDSs; (4) rRNA and tRNA; (5) GC content; and (6) GC skew value(G − C/G + C). (**E**) COG functional annotation and categorization of genome-encoded proteins. (**F**) The genomic annotation of strain NND9 via the GO database. (**G**) The genomic annotation of strain NND9 via the KEGG database.

Preliminary screening for bacterial isolates with protective activities on colonic epithelium indicated strain NND9 as one of the most possible candidates, so we carried out morphological and genomic characterization on it. NND9 made off-white, circular, raised, translucent colonies on a BHI agar plate containing mucin (Figure 1A). Under the scanning electron microscope, the cells were oval or rod-shaped, about 0.4–0.6 µm in diameter and 0.8–1.2 µm in length, without flagella or other appendages (Figure 1B). Based on 16S rDNA analysis, NND9 was most closely related to *Akkermansia muciniphila* and clustered on the same evolutionary branch of the phylogenetic tree as the type strain *A. muciniphila* ATCC BAA-835 (Figure 1C). Therefore, we designated the strain as *Akkermansia muciniphila* NND9. The genome of NND9 is 2,737,899 bp in length, containing 2646 CDSs, 53 tRNAs, 9 rRNAs, and 77 CRISPR repeats (Figure 1D). The genomes of NND9 and *A. muciniphila* ATCC BAA-835 shared high similarity by nucleotide sequence (99% similarity) and G + C content (55.80% for ATCC BAA-835 and 55.67 for NND9). The predicted proteins were functionally annotated and classified through comparison with the COG database, with those involved in cell wall/membrane/envelope biogenesis being the most abundant (Figure 1E). GO annotation of NND9 coding genes revealed gene product distributions across cellular components, molecular functions, and biological processes, with the largest number of genes being enriched in cellular processes (Figure 1F). KEGG annotation analysis of the coding genes of NND9 indicated genes involved in metabolic pathways being the most abundant (Figure 1G).

### 3.2. NND9 Alleviates DSS-Induced Ulcerative Colitis in Mice

**Figure 2:**
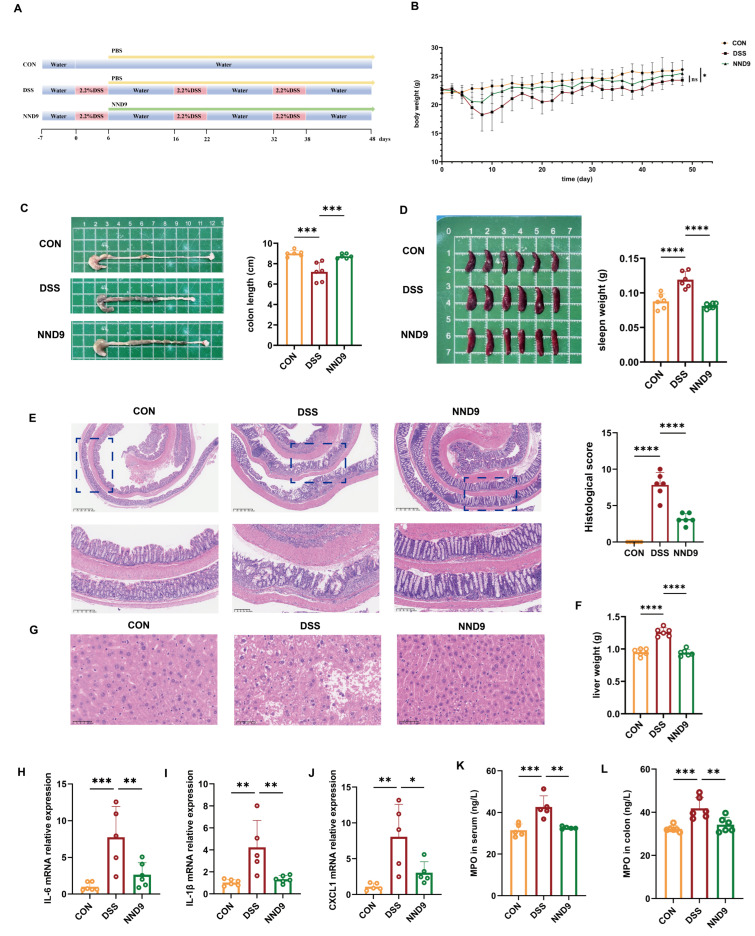
NND9 alleviates DSS-induced chronic ulcerative colitis in mice. (**A**) Animal experimental procedure. (**B**) Daily body weight changes in mice (n = 6 per group). (**C**) Representative colon images (left) and quantitative analysis of colon length changes. (**D**) Representative spleen size images (left) and spleen weight (right). (**E**) Representative H&E-stained colon tissue sections and histopathological scoring. Scale bars: 625 µm (top) and 200 µm (bottom). (**F**) Changes in mice liver tissue weight. (**G**) Representative H&E-stained images of the mouse liver tissue sections. Scale bars: 50 µm. (**H**–**J**) Relative mRNA expression levels of IL-6, IL-1β, and CXCL1, respectively, in the mouse colon tissue. (**K**,**L**) MPO activity in the mouse serum and colon, respectively, before (DSS) and after NND9 treatment. * *p* ≤ 0.05, ** *p* ≤ 0.01, *** *p* ≤ 0.001, **** *p* ≤ 0.0001 and ns means *p* > 0.05.

To assess the protective effects of NND9 on ulcerative colitis (UC), we established a DSS-induced UC mouse model and orally administered live *A. muciniphila* NND9 to the mice (Figure 2A). NND9 alleviated weight loss in mice treated with DSS, although the difference was not statistically significant (Figure 2B). Additionally, NND9 markedly reduced DSS-induced colon shortening and splenomegaly (Figure 2C,D). DSS-treated mice exhibited severe mucosal damage, characterized by disorganized crypt arrangement, markedly reduced crypt number, evident gut epithelial injury, and significant inflammatory cell infiltration; NND9 supplementation increased crypt number and height while mitigating gut epithelial damage (Figure 2E). In addition, compared with the controls, mice treated with DSS had a significantly increased liver weight, and NND9 administration restored the normal liver weight (Figure 2F). Histological examinations revealed pronounced hepatocyte damage in mice of the DSS group, while treatment with NND9 reduced hepatocyte damage and improved the integrity of the liver tissue (Figure 2G).

We further evaluated the treatment effects of NND9 on inflammatory mediators and found that NND9 treatment significantly decreased the expression levels of representative proinflammatory IL-6 (cytokine interleukin-6), IL-1β (interleukin-1β), and the chemokine CXCL1 in the colonic tissue (Figure 2H–J). Additionally, NND9 treatment also downregulated myeloperoxidase (MPO) in both the colonic tissue and serum (Figure 2K,L). MPO causes increased production of reactive oxygen species (ROS), which can directly damage surrounding tissues and activate a series of inflammatory signaling pathways. Our results indicate that NND9 mitigated the experimental UC by resolving the inflammatory responses.

### 3.3. NND9 Enhances the Gut Mucosal Barrier Function in UC Mice

**Figure 3:**
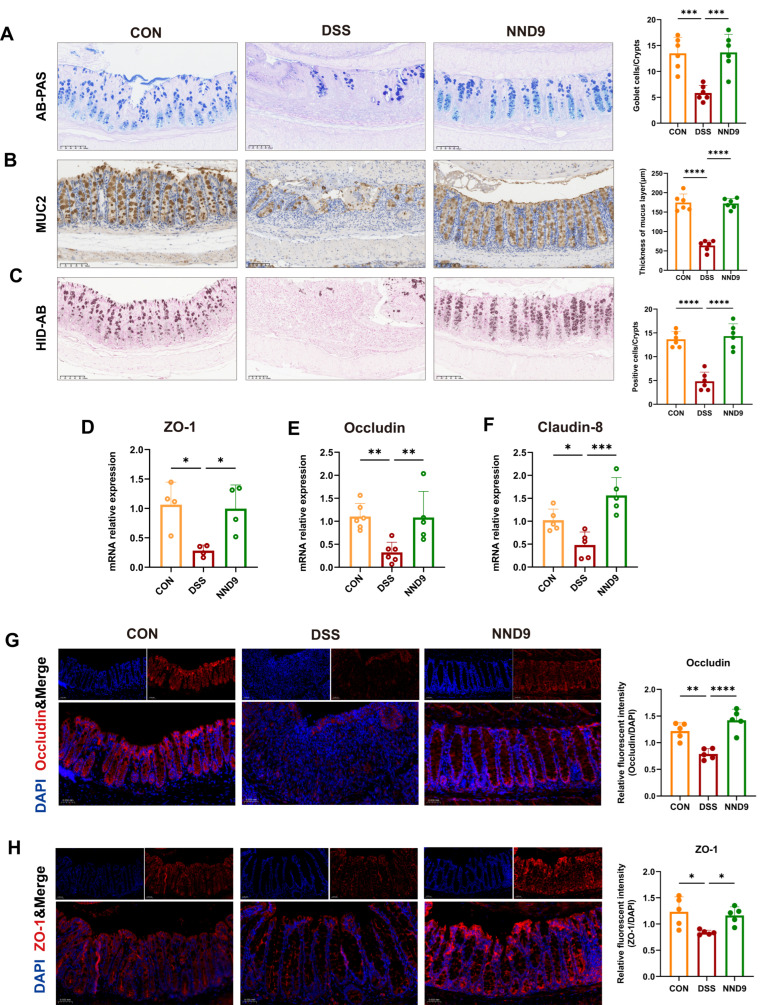
NND9 treatment enhances the mucosal barrier function in the mouse colonic tissue. (**A**) Representative image of AB&PAS staining (mucous substances appear blue or purple) (left) and quantitative analysis of goblet cells (right). Scale bars: 100 µm. (**B**) Representative microscopic image of MUC2 immunohistochemical staining (MUC2 positive cells appear brown) (left) and quantitative analysis of mucus layer thickness (right). Scale bars: 100 µm. (**C**) Representative image of HID-AB staining in the mouse colon tissue (left; sulfated mucins appear mahogany) and counting analysis (right). Scale bars: 100 µm. (**D**–**F**) Relative mRNA expression levels of ZO-1, Occludin and Claudin-8, respectively, in the colon of the mice by quantitative RT-PCR. (**G**) Immunofluorescence staining images of Occludin (red) and DAPI (blue) in colon tissue and quantification of the relative fluorescence intensity. Scale bars: 500 µm. (**H**) Immunofluorescence staining microscopic image of ZO-1 (red) and DAPI (blue) in colon tissue and quantification of the relative fluorescence intensity. Scale bars: 500 µm. * *p* ≤ 0.05, ** *p* ≤ 0.01, *** *p* ≤ 0.001, **** *p* ≤ 0.0001.

Maintaining the integrity of the gut barrier is crucial for delaying the progression of UC. Goblet cells (GCs) on the colonic surface continuously secrete Muc2 to maintain the integrity of the mucus layer and chronic inflammation can lead to the loss of GCs within the mucosal layer. The UC mice had severe GCs depletion in the colonic tissue and the administration of NND9 markedly ameliorated this condition (Figure 3A,B). Muc2 comprises distinct subtypes, some of which may undergo sulfation modification. Elevated levels of sulfated mucin in the colon help prevent bacterial enzymes from degrading mucin glycans, thereby effectively blocking close contact between harmful factors and the gut epithelium [17,27,28]. In our UC model mice, the sulfated mucin in the colonic tissue was radically reduced, while administration of NND9 recovered its levels (Figure 3C). These findings demonstrate that NND9 could inhibit the progression of colitis by influencing the biochemistry in the mucus layer.

Tight junction proteins, such as ZO-1, occludin and claudin, constitute the core protein complex forming intercellular tight junctions and play a crucial role in maintaining the gut barrier [29,30]. In the colitis mice, the mRNA expression of ZO-1, occludin and claudin-8 was significantly reduced and NND9 treatment recovered the expression levels (Figure 3D–F); immunofluorescence staining revealed the same effects of NND9 treatment at the protein level (Figure 3G,H). These results demonstrate that NND9 maintained the gut barrier function by reducing GCs depletion, increasing mucin secretion, and enhancing tight junction protein expression, thereby alleviating DSS-induced chronic colitis in the mice.

### 3.4. NND9 Modulates Gene Expression in the Colon Tissue During DSS-Induced Colitis

**Figure 4:**
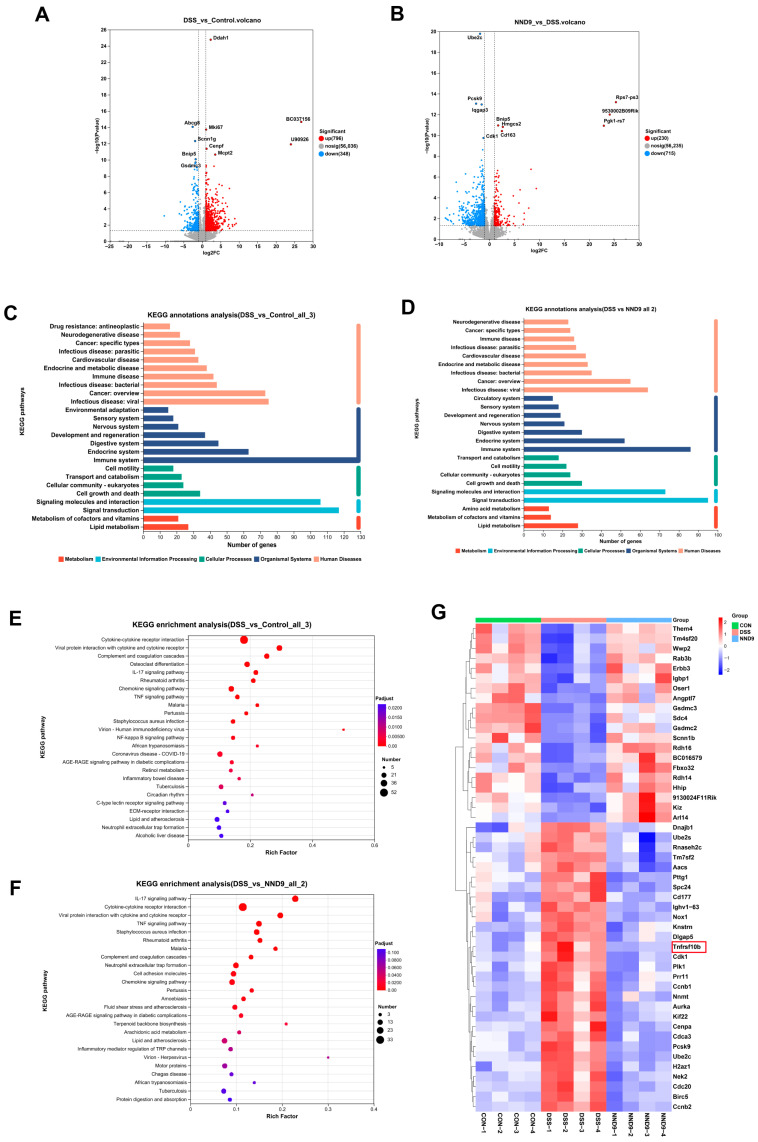
Transcriptomic analysis of colon tissue following NND9 treatment in UC mice. (**A**) The volcano plot displays the relative changes in gene expression between Control mice and DSS-induced mice in colon tissue (DSS vs. control). (**B**) The volcano plot displays the relative changes in gene expression in the colon tissue of mice following NND9 treatment (NND9 vs. DSS). (**C**,**D**) KEGG functional annotation analysis of differentially expressed genes in the mouse colon tissue, with (**C**) showing the differentially expressed genes in the DSS and control groups and (**D**) showing the differentially expressed genes in the NND9 and DSS groups. (**E**,**F**) KEGG functional enrichment analysis of differentially expressed genes in the mouse colon tissue, with (**E**) displaying the differentially expressed gene in the DSS group and the control group and (**F**) displaying the differentially expressed gene in the NND9 group and the DSS group. (**G**) Heatmap showing differential gene clustering analysis of colon tissues from control, DSS, and NND9 mice. Screening criterion: *p* ≤ 0.001.

To investigate the potential mechanisms by which NND9 protects the gut barrier, we performed transcriptomic analysis on the colon tissue and identified changes in gene expression among mice of different groups (Figure 4A,B). We then conducted functional enrichment and annotation analyses on the differentially expressed genes (DEGs) and profiled overall RNA expression alterations of the colonic tissues in the mice following DSS modeling and NND9 treatment. KEGG annotation analysis showed that the DEGs were mainly involved in immune functions, cell growth and death, signal transduction, and lipid metabolism (Figure 4C,D). KEGG enrichment analysis of the DEGs between mice of the DSS and NND9 groups revealed significant enrichment in inflammatory signaling pathways, including IL-17, TNF and chemokine signaling pathways, as well as the cytokine-cytokine receptor interaction pathways (Figure 4E,F). In order to identify potential targets of the NND9 metabolites, we performed a clustering analysis of the DEGs among mice of the CON, DSS and NND9 groups. The heatmap revealed that NND9 significantly influenced genes associated with cell survival or proliferation. Notably, we observed a marked suppression of Tnfrsf10b gene expression in the colonic tissue following NND9 administration (Figure 4G).

### 3.5. NND9 Protects the Gut Barrier by Inhibiting DR5-Mediated Epithelial Cell Death

**Figure 5:**
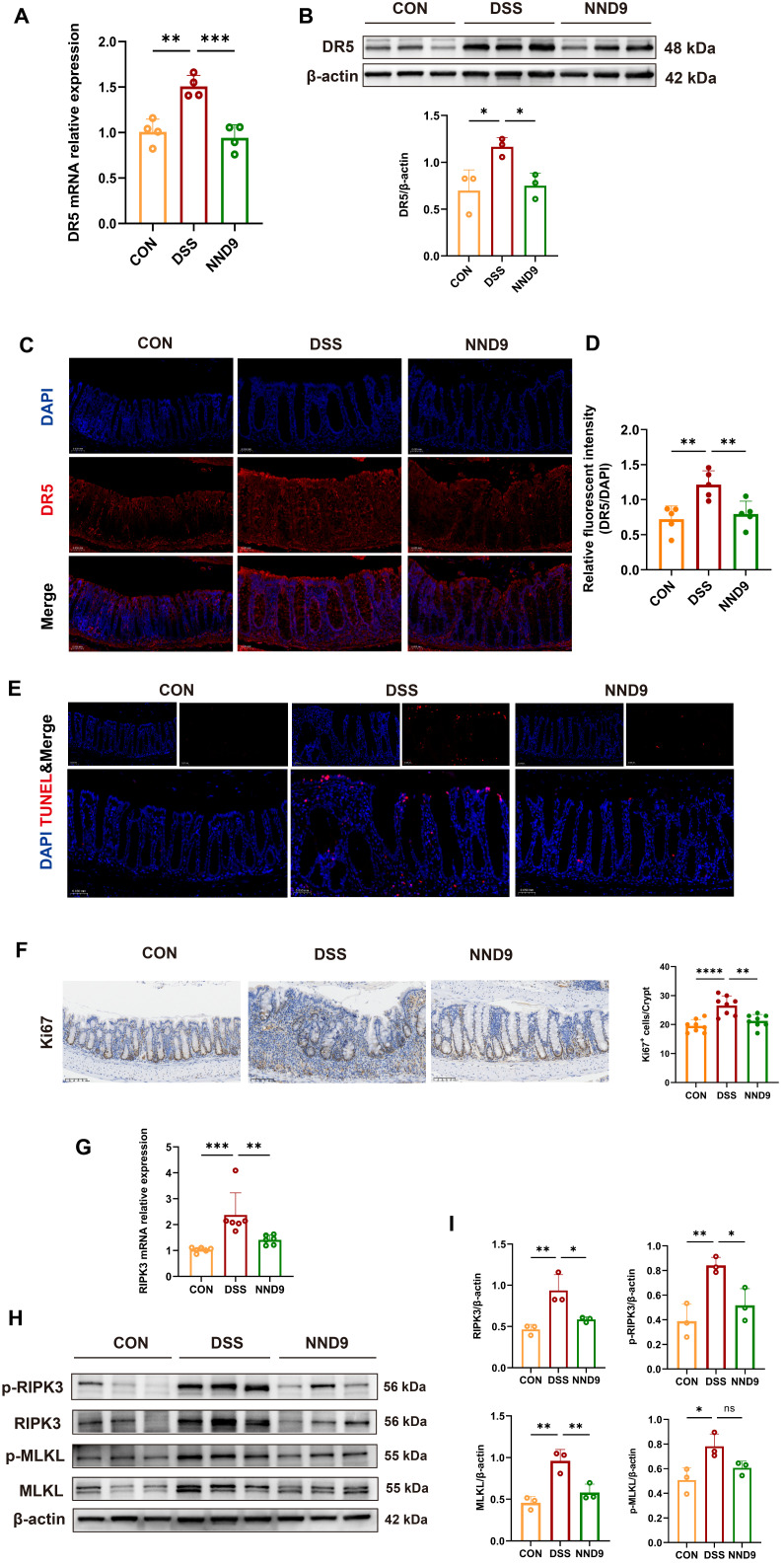
NND9 protects the gut epithelial barrier by inhibiting epithelial cell death through reduced expression of DR5 protein in colonic tissue. (**A**) Relative mRNA expression levels of DR5 in the mouse colon tissue. (**B**) The protein expression of DR5 in the colon of mice in different groups by Western blot and quantification of the value of DR5/β-actin. (**C**) Immunofluorescence staining of DR5 (red) and DAPI (blue) in the mouse colon tissue. Scale bars: 500 µm. (**D**) Quantitative analysis of relative fluorescence intensity of DR5 protein. (**E**) TUNEL fluorescence staining. Red areas indicate TUNEL-positive cells. Scale bars: 500 µm. (**F**) Representative image of Ki67 immunohistochemical staining in colon tissue (left) and quantitative analysis of Ki67-positive cells (right). Scale bars: 100 µm. (**G**) Relative mRNA expression levels of RIPK3 in the mouse colon tissue. (**H**) Western blot analysis of necroptosis-related proteins in colon tissue from different groups of mice. (**I**) Quantitative analysis of protein levels and relative expression of necrotic apoptosis markers in colon tissue from different groups of mice. * *p* ≤ 0.05, ** *p* ≤ 0.01, *** *p* ≤ 0.001, **** *p* ≤0.0001 and ns means *p* > 0.05.

*Tnfrsf10b*, also known as Tumor necrosis factor receptor superfamily member 10b or Death receptor 5 (DR5), is a transmembrane receptor located on the cell membrane. It induces cell death by binding to TNF-related apoptosis-inducing ligand (TRAIL) and activating downstream signaling pathways [19,20,31]. We first validated via RT-PCR that NND9 administration suppressed the increase in DR5 mRNA expression in the colonic tissue of the DSS-treated mice (Figure 5A). Western blot and immunofluorescence analyses demonstrated that NND9 reduced the protein expression of DR5 in the colonic tissues of the DSS-treated mice (Figure 5B–D) and alleviated gut epithelial cell death (Figure 5E). Concurrently, the number of Ki67-positive cells increased in DSS-treated colonic tissue, likely as a compensatory response to make for the DR5-associated cell death by increased gut epithelial cell proliferation, and NND9 redressed the condition (Figure 5F).

Research has shown that the gut epithelial cell necrosis promotes the progression of colitis [32]; DR5 activation not only initiates apoptosis but also triggers necrotic cell death [21,22,33]. Therefore, we further investigated whether DR5 receptor activation on the surface of gut epithelial cells in colitis mice could initiate the necrotic apoptosis pathway, and whether NND9 could reduce gut epithelial cell death by regulating the epithelial necrotic apoptosis pathway. As the phosphorylation of RIPK3 and MLKL plays a crucial role in necrotic apoptosis, we first detected the mRNA expression of RIPK3, a key protein in this pathway, via RT-PCR. The results showed that RIPK3 mRNA expression was significantly elevated in the DSS group, while NND9 administration markedly suppressed RIPK3 expression (Figure 5G). Consistently, immunoblotting results showed that, following DSS treatment, the expression levels of P-RIPK3, P-MLKL, RIPK3, and MLKL were significantly increased in the colon tissues of colitis model mice; NND9 administration effectively suppressed the expression of these molecules (Figure 5H,I). Taken together, these findings indicate that NND9 reduces gut epithelial cell death by modulating the necrotic apoptosis pathway, thereby repairing the gut barrier and alleviating colitis in the UC model mice.

### 3.6. NND9 Downregulates DR5 Expression of the Gut Epithelial Cells and Suppresses Excess Cell Death

**Figure 6:**
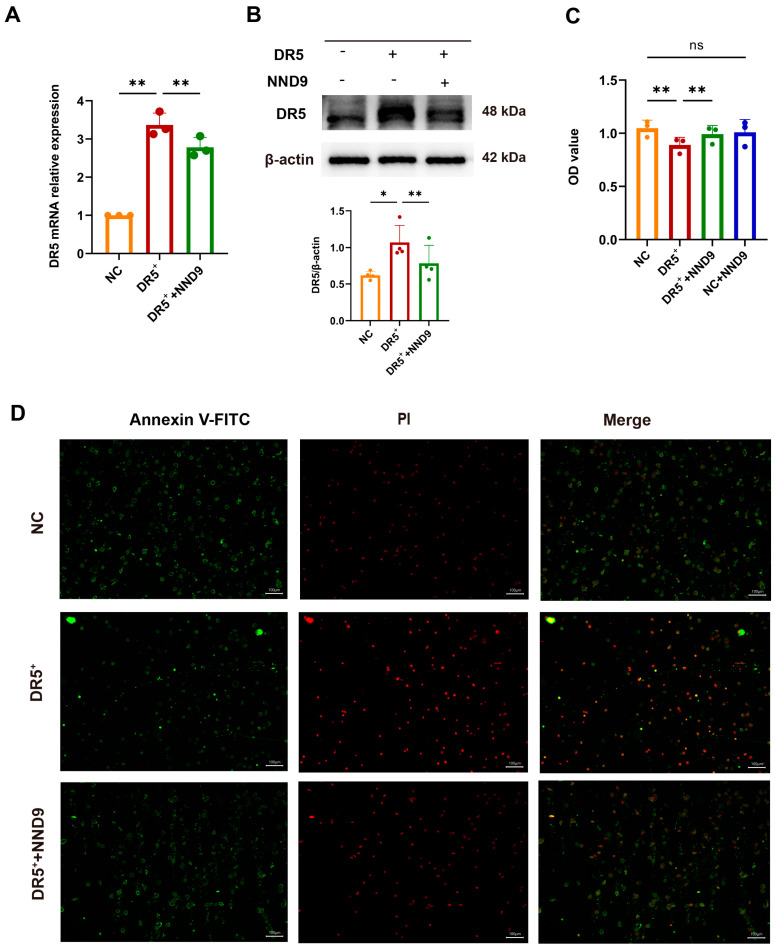
NND9 downregulates DR5 expression of gut epithelial cells and suppresses excess cell death. (**A**) The relative mRNA expression levels of DR5 in NCM460 cells by quantitative RT-PCR. (**B**) Protein expression of DR5 in NCM460 cells by Western blot and quantification of the value of DR5/β-actin. (**C**) Cell viability test. (**D**) Immunostaining images of NCM460 cells stained for Annexin V-FITC (green, early apoptotic cells), PI (red, late apoptotic or necrotic cells), and merge. Scale bars: 100 µm. * *p* ≤ 0.05, ** *p* ≤ 0.01 and ns means *p* > 0.05.

To determine whether NND9 can reduce the expression of the DR5 protein in gut epithelial cells, we overexpressed the DR5 gene on NCM460 cells and confirmed that NND9 treatment reduced the overexpression of DR5 mRNA and protein in NCM460 cells (Figure 6A,B). We further found that, compared with the NC group, the overexpression of DR5 impaired cell proliferative capacity of NCM460 cells, which was regained by NND9 treatment (Figure 6C). Additionally, DR5 overexpression led to marked increase in the number of Annexin V-FITC and PI double-positive cells, indicating that cells with DR5 overexpression exhibited characteristics of necroptosis. Notably, NND9 treatment reduced the number of PI-positive cells in NCM460 cells (Figure 6D). These results indicate that NND9 could reduce the death of the gut epithelial cells by decreasing the expression of DR5 protein in these cells.

### 3.7. NND9 Ameliorates the Gut Microbiome in DSS-Induced Ulcerative Colitis Mice

**Figure 7:**
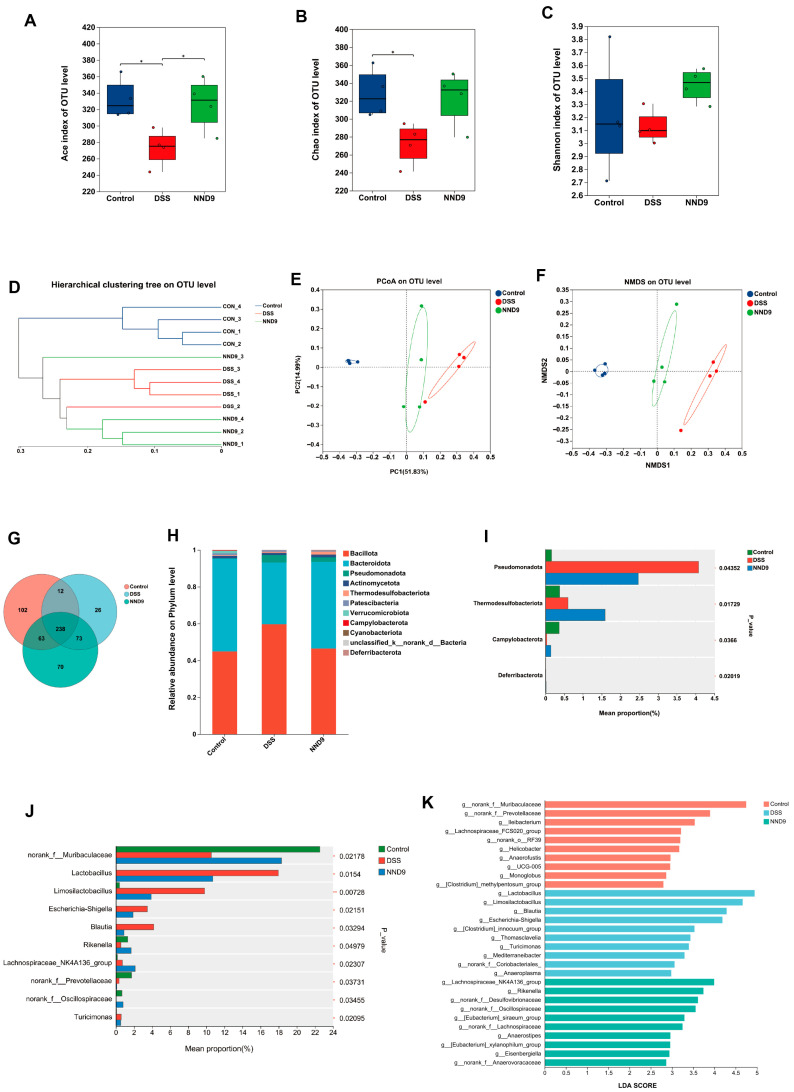
NND9 modulates the gut microbiota composition in DSS-induced UC mice. (**A**–**C**) Analysis of Alpha diversity in the gut microbiota showing (**A**) Ace index, (**B**) Chao index and (**C**) Shannon index reflecting gut microbial species diversity. (**D**–**F**) Beta diversity analysis of the gut microbiota, showing (**D**) hierarchical cluster of fecal samples at the OTU level, (**E**) PCoA demonstrating similarities and differences between samples, and (**F**) NMSD analysis at the OUT level among different fecal samples. (**G**) The species Venn diagram analysis of fecal samples reveals shared and unique species among different groups of mice. (**H**) Phylum-level taxonomic composition of fecal microbiomes. (**I**) Differential analysis of species abundance at the phylum level among different groups. (**J**) Genus-level microbial composition differences among groups of mice. (**K**) The linear discriminant analysis of effect size. * *p* ≤ 0.05.

To investigate the ameliorating effects of NND9 on the gut microbiota in the colitis mice, we collected the fecal samples before and after NND9 treatment and profiled changes in the composition of the gut microbiota by 16S rDNA sequencing. DSS induced colitis in the mice and in the meantime also caused gut microbiota dysbiosis; the decreased ACE and Chao indices of species richness, reflecting the alpha diversity of gut microbiota, were restored by NND9 treatment (Figure 7A,B). Meanwhile, the Shannon index revealed that the reduced gut microbial species diversity in DSS-treated mice was recovered by NND9 treatment (Figure 7C). To investigate the similarities or differences in overall gut microbiota community structure after intervention with NND9, we conducted sample-level clustering analysis, PCOA analysis, and NMSD analysis at the OTU level. Very interestingly, whereas mice of the DSS group and the CON group exhibited distinct microbial clustering, NND9 treatment brought the clustering toward that of the CON group, demonstrating that NND9 can modulate the overall community structure of the gut microbiota in the colitis mice (Figure 7E,F).

To profile the bacterial taxa involved in the gut ecological changes in colitis modeling by DSS and in the amelioration by NND9, we analyzed the phylogenetic composition of the fecal microbiota across the groups (Figure 7G). At the phylum level, the gut microbiota was dominated by Bacillota and Bacteroidota. Following DSS treatment, the abundance of Bacillota significantly increased in colitis mice, while the Bacteroidota phylum decreased (Figure 7H). An increase or decrease in the ratio between these two phyla is often regarded as an ecological imbalance [34]. Administration of NND9 reduced the Bacillota/Bacteroidota ratio, indicating that NND9 alleviated the DSS-induced gut dysbiosis. Additionally, compared to the CON group, DSS-treated mice exhibited increased Pseudomonadota abundance; NND9 administration reduced Pseudomonadota abundance (Figure 7I). To gain deeper insights into the compositional changes in the gut microbiota during the chronic UC disease process and NND9 intervention, we conducted multiple differential abundance tests and found that, after DSS treatment, four bacterial genera, i.e., *Lactobacillus*, *Limosilactobacillus*, *Escherichia-Shigella*, and *Blautia,* were enriched, while Muribaculaceae genera and *Rikenella* were reduced (Figure 7J). We performed LefSe (the linear discriminant analysis of effect size) analysis and found that the LDA score plot displayed significantly differential genera across the three groups (Figure 7K). LDA scores represent the magnitude of contribution from differential groupings, aiding in the identification of microbes potentially playing roles during colitis or recovery from the disease.

### 3.8. NND9 Modulates Metabolism of Gut Bacteria to Mitigate DSS-Induced Colitis in the Mice

**Figure 8:**
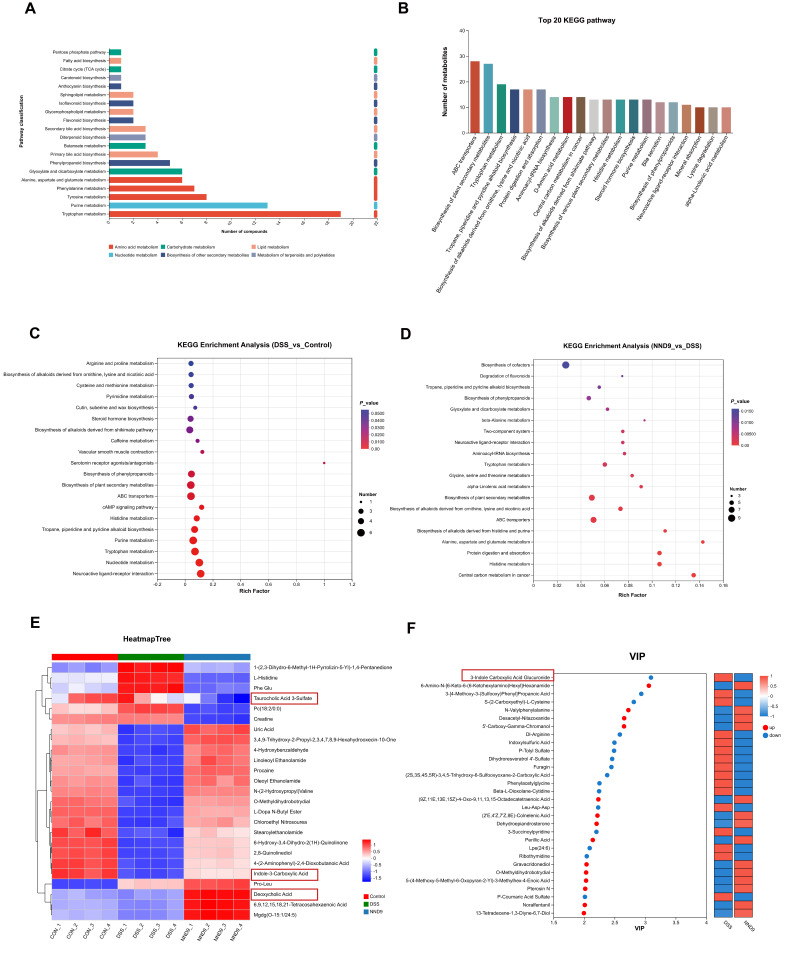
Untargeted metabolomics analysis of the mouse fecal samples. (**A**,**B**) KEGG functional annotation of all identified compounds in fecal samples from different groups. Metabolic pathways involved in different metabolic pathway categories (**A**) and metabolic pathways ranked by the number of metabolites involved in them (top 20) (**B**). (**C**) KEGG enrichment analysis bubble plot of differentially expressed metabolites in fecal samples from the DSS group and the control group. (**D**) KEGG enrichment analysis bubble plot of differentially expressed metabolites in NND9 and DSS fecal samples. (**E**) Clustering heatmap of fecal differential metabolites across groups. (**F**) VIP bubble plot and expression heatmap of differential metabolites in NND9 and DSS fecal samples.

Multiple studies have demonstrated that changes in the gut microbiome and metabolome are closely associated with the development of colitis [35]. Gut microbiota-derived metabolites serve as key mediators in gut microbiota–host interactions [36]. Certain gut microbial metabolites are known to play pivotal roles in maintaining or disrupting gut homeostasis, thereby alleviating or exacerbating colitis [36,37,38]. To recognize the bacteria and their metabolites that are involved in healing or promoting colitis, we performed untargeted metabolomic analyses of the mouse fecal samples and annotated all identified compounds using the KEGG database. The relevant metabolic pathways included tryptophan metabolism, purine metabolism, primary bile acid biosynthesis, secondary bile acid biosynthesis, and fatty acid biosynthesis (Figure 8A,B). We further performed KEGG pathway enrichment analysis on differentially expressed metabolites among the groups of mice and identified tryptophan metabolism, purine metabolism, and cysteine and methionine metabolism as the primary enriched pathways (Figure 8C,D). To uncover the metabolites potentially playing a key role in alleviating colitis, we performed cluster analysis on differential metabolites between the DSS-treated and NND9-treated mice. As shown on the heatmap tree, administration of NND9 restored the abundance of indole-3-carboxylic acid in the DSS-treated mice (Figure 8E). Concurrently, VIP analysis revealed significantly reduced abundance of 3-indole carboxylic acid glucuronide in NND9 group fecal metabolites, suggesting that it is potentially deconjugated by gut microbial β-glucuronidase to form free indole-3-carboxylic acid (Figure 8F). Indole compounds are metabolites produced through microbial metabolic pathways from tryptophan [39,40]. Previous studies indicate that indole-3-carboxylic acid can improve gut barrier damage and suppress the production of inflammatory cytokines IL-6, IL-1β and TNF-α [41]. Therefore, our findings suggest that NND9 may alleviate colitis by regulating indole-3-carboxylic acid production.

Additionally, NND9 significantly reduced the abundance of taurocholic acid 3-sulfate, a primary bile acid derivative, while markedly increasing the abundance of deoxycholic acid (DCA), a secondary bile acid (Figure 8F). Secondary bile acids are produced in the gut through the metabolic modification of primary bile acids by gut bacteria and our results indicate that NND9 mediated the biotransformation of bile acids by the gut microbiota. Previous studies have demonstrated bidirectional effects of DCA on colitis. In DSS-induced mouse colitis models, DCA alleviates disease severity. Rectal supplementation with DCA reduces DSS-induced colonic loss and mucosal damage while decreasing inflammatory cytokine production [42]. These results suggest that NND9 may enhance DCA production by modulating gut microbiota composition to exert anti-inflammatory effects and alleviate colitis.

## 4. Discussion

In this study, we aimed at investigating the curative roles of *A. muciniphila* NND9 in ulcerative colitis, with a focus on its molecular mechanisms in protecting the gut barrier. The results demonstrated that NND9 can alleviate colitis severity in the mouse model and protect the gut barrier. Specifically, NND9 alleviated goblet cell depletion and promoted mucus production, thereby restoring the thickness of the mucosal layer. NND9 also enhanced the expression of tight junction proteins and reduced gut epithelial cell death by suppressing DR5 expression on the cell surface. Additionally, NND9 restored gut homeostasis and gut microbiota metabolism. These findings demonstrate that *A. muciniphila* NND9 played beneficial roles in mitigating colitis.

A growing body of research indicates that the gut microbiome plays significant roles in the onset and progression of UC [43,44]. For example, *A. muciniphila* could alleviate DSS-induced ulcerative colitis in mice [45], but much has remained unclear about the mechanisms by which *A. muciniphila* protects the colonic epithelial barrier. Building upon our team’s previous research, we observed increased *Akkermansia* genus abundance in the feces of mice with chronic ulcerative colitis induced by DSS. Therefore, we sought to investigate whether *Akkermansia* strains isolated from diseased states have the same ulcerative colitis-alleviating effects and to explore the underlying mechanisms of *Akkermansia* strains. Of enormous interest, we isolated the UC-mitigating *Akkermansia* strain, *A. muciniphila* NND9, from a progressive UC mouse model, which suggests that, while UC is progressing, healing processes are also potentially going on. Once the UC pathogenic actions and the healing reactions reach some kind of equilibrium, a beneficial intervention, such as amelioration of the gut microbiome, may enhance *A. muciniphila* in population size or functionality or both, tilting the disease condition toward recovery. Variations in *Akkermansia* abundance have been reported among different UC patients and mouse models, with some reports implicating pathogenic roles of *Akkermansia* [9]. Our findings in this study demonstrate that the *Akkermansia* lineage represented by NND9 played protective roles in experimental UC, suggesting that variations in *Akkermansia* abundance among patients or animals may reflect different stages of the pathological/healing processes or different lineages of *Akkermansia* detected. Bacteria as closely related as sharing >99% 16S rDNA sequence similarity may have radically different biological properties, such as one having potent anticancer activities but another being totally incompetent, as they may belong to distinct natural species isolated by genetic boundaries [46,47].

NND9 presented remarkable curative effects on UC by reducing mucosal damage and lowering the expression of inflammatory markers, including IL-1β, IL-6, and MPO (See Figure 2). The gut mucus layer serves as a critical frontline barrier isolating gut microbiota or harmful substances from the gut epithelium and, additionally, the biochemical state of mucins influences colonic barrier function [48]. In healthy individuals or mice, sulfated mucins predominate in the gut to maintain the epithelial barrier functionality. Multiple studies have indicated that reduced sulfated mucin content increases susceptibility to colitis and enhances bacterial invasion of the gut barrier [49,50,51]. In our study, DSS challenge reduced sulfated mucin content in the mice, and administration of NND9 increased sulfated mucin levels in the gut mucus layer (See Figure 3). Underneath the mucus layer is the gut epithelium, which is the structural barrier that keeps foreign antigens, inflammatory factors, and other toxicants in the gut lumen from entering the bloodstream and deep tissues. Tight junctions bring adjacent epithelial cells into close contact, sealing the intercellular spaces and forming a physical barrier structure surrounding the cells [52]. Our experimental results demonstrated that NND9 significantly enhanced the mRNA expression levels of tight junction proteins Cldn8, Occludin, and ZO-1 in colitis mice, suggesting that NND9 can strengthen tight junctions, improving gut barrier integrity and reducing intestinal permeability.

Of great significance, DR5 expression at both mRNA and protein levels was markedly elevated in the colonic tissue of DSS-treated mice, and NND9 significantly reduced the expression levels. As the activation of DR5 protein can induce apoptosis and necrotic cell death processes, we hypothesized that its overexpression may lead to excessive epithelial cell death, thereby disrupting the gut epithelial barrier. Analysis by TUNEL staining revealed a significant increase in cell death within colonic tissues of the DSS group, whereas NND9 effectively reduced epithelial cell death (See Figure 5). Additionally, our results revealed elevated phosphorylation levels of RIPK3 and MLKL, which are key proteins in the necrotic apoptosis pathway, in the colonic tissues of UC mice and NND9 effectively suppressed the expression of p-RIPK3 and p-MLKL proteins (See Figure 5), indicating that NND9 suppressed the necrotic apoptosis pathway in the colon tissue of UC mice. Although the specific roles and mechanisms of cell necrosis and apoptosis in chronic colitis remain poorly understood [53,54,55], numerous studies have already initiated research on the elucidation of the necrosis apoptosis pathway and mechanisms to alleviate colitis [56,57]. We provided evidence in this study, showing that inhibiting the overexpression of DR5 protein in gut epithelial cells during colitis may suppress cell death pathways, thereby reducing epithelial cell death and preserving the gut epithelial barrier.

The gut microbiome participates in gut immune responses during colitis, and host–microbe interactions play a crucial role in the development of colitis. Under normal physiological conditions, the gut symbionts produce beneficial metabolites that maintain homeostasis. However, during suboptimal health or disease states, potentially harmful microorganisms may occupy ecological niches, becoming dominant species that disrupt gut ecological stability. Therefore, preserving gut ecological homeostasis represents an effective strategy to inhibit colitis progression. Previous studies have demonstrated reduced diversity of gut microbial taxa in UC patients [58]. Consistent with the previous reports, our experimental results demonstrate reduced α-diversity in the gut microbiota of chronic UC mice, while NND9 can restore the gut microbial diversity in these animals. Notably, the abundance of bacteria in the Muribaculaceae family and *Rikenella* genus was significantly reduced in the UC mice and NND9 helped recover the abundance of these bacteria. These bacteria produce short-chain fatty acids and enhance mucin regeneration in colonic tissue, thereby playing beneficial roles in alleviating colitis [59]. These findings demonstrate that NND9 can restore gut microbiota structure in colitis mice and modulate gut ecological imbalance.

Gut microbial metabolites serve as mediators of microbe–host interactions. In our study, the abundance of indole-3-carboxylic acid (ICA) in the feces of UC mice was significantly reduced, and NND9 regained the abundance (See Figure 8). A recent study reports that ICA can stimulate IL-22 secretion by activating the nuclear receptor Roryt in gut type 3 innate lymphoid cells (ILC3s), thereby repairing gut barrier damage and enhancing its protective function. NND9 can also increase the abundance of the secondary bile acid deoxycholic acid (DCA) while decreasing the abundance of primary bile acid derivatives to mitigate UC severity. Bile acid metabolism is a microbe-mediated pathway within the gut, where primary bile acids are modified by gut microbiota in the colon to form secondary bile acids. Primary bile acids accumulate and secondary bile acids decrease in IBD patients as a result of reduced gut microbial diversity, which impedes bile acid metabolism pathways [60,61]. Our experimental results align with this. In summary, our findings suggest that NND9 regulated the gut homeostasis by modulating indole compound metabolism and the bile acid metabolic pathways.

## 5. Conclusions

In conclusion, our study demonstrates that *A. muciniphila* NND9 alleviates ulcerative colitis in mice by reducing inflammation, protecting the intestinal mucus barrier and the intestinal epithelial barrier, and modulating intestinal dysbiosis and gut metabolism. Importantly, our study reveals DR5 as a potential therapeutic target for alleviating chronic UC. Collectively, these findings addressed the crucial role of *Akkermansia muciniphila* strain NND9 in maintaining intestinal homeostasis, positioning it as a potential novel agent for modulating chronic colitis.

## Figures and Tables

**Table 1:** The criteria used for histopathological scoring.

Severity of Inflammation	Inflammation Infiltration	Epithelial/Crypt Damage
0 = none	0 = none	0 = none
1 = mild	1 = mucosa	1 = basal 1/3
2 = moderate	2 = mucosa and submucosa	2 = basal 2/3
3 = severe	3 = transmural	3 = crypt loss
		4 = crypt and surface epithelial destruction

**Table 2:** Primer sequences used for real-time PCR.

Gene Name		Primer Sequence (5′-3′)
mACTB	Forward	GATATCGCTGCGCTGGTCG
	Reverse	CATTCCCACCATCACACCCT
mIL-6	Forward	CCAAGAGGTGAGTGCTTCCC
	Reverse	CTGTTGTTCAGACTCTCTCCCT
mIL-1β	Forward	AATGCCACCTTTTGACAGTGAT
	Reverse	ATCAGGACAGCCCAGGTCAA
mCxcl1	Forward	CTGGGATTCACCTCAAGAACATC
	Reverse	CAGGGTCAAGGCAAGCCTC
mOccludin	Forward	TTGAAAGTCCACCTCCTTACAGA
	Reverse	CCGGATAAAAAGAGTACGCTGG
mZO-1	Forward	GCCGCTAAGAGCACAGCAA
	Reverse	GCCCTCCTTTTAACACATCAGA
mClaudin-8	Forward	AAGGTCTACGACTCCCTGCT
	Reverse	CATTCCGAGGATGGCTGTCA
mTnfrsf10b	Forward	GCGAACTCTGTGCATTCGTC
	Reverse	TCGTCAGCTGAGTCGTTTCC
mRipk3	Forward	ACCCCACCGAATCCAATGAC
	Reverse	GCCGAACTTGAGGCAGTAGT
hACTB	Forward	ACCGCGAGAAGATGACCCAG
	Reverse	GGATAGCACAGCCTGGATAGCAA
hTnfrsf10b	Forward	TCCCTGTTCTCTCTCAGGCA
	Reverse	CCAGGTCGTTGTGAGCTTCT

## Data Availability

All data and resources are available upon request. The raw data reported in this paper have been deposited in the China National Center for Bioinformation (CNCB)/Beijing Institute of Genomics, Chinese Academy of Sciences. The whole genome sequence data are publicly accessible at https://ngdc.cncb.ac.cn/gsa (accessed on 9 February 2026): accession no. CRA038441. The RNA-seq data are accessible at https://ngdc.cncb.ac.cn/gsa: accession no. CRA038372. The metagenome data are archived at https://ngdc.cncb.ac.cn/gsa: accession no. CRA038391. The metabolome data are available at https://ngdc.cncb.ac.cn/omix (accessed on 9 February 2026): accession no. OMIX015041.
